# Supplementary material for: Deep Learning Predicts Postoperative Mobility, Activities of Daily Living, and Discharge Destination in Older Adults from Sensor Data
Source: Sensors (Basel). 2025 Aug 13;25(16):5021. doi: 10.3390/s25165021 (PMC12389988; doi:10.3390/s25165021)

Supplementary Figures

Figure S1: STROBE chart

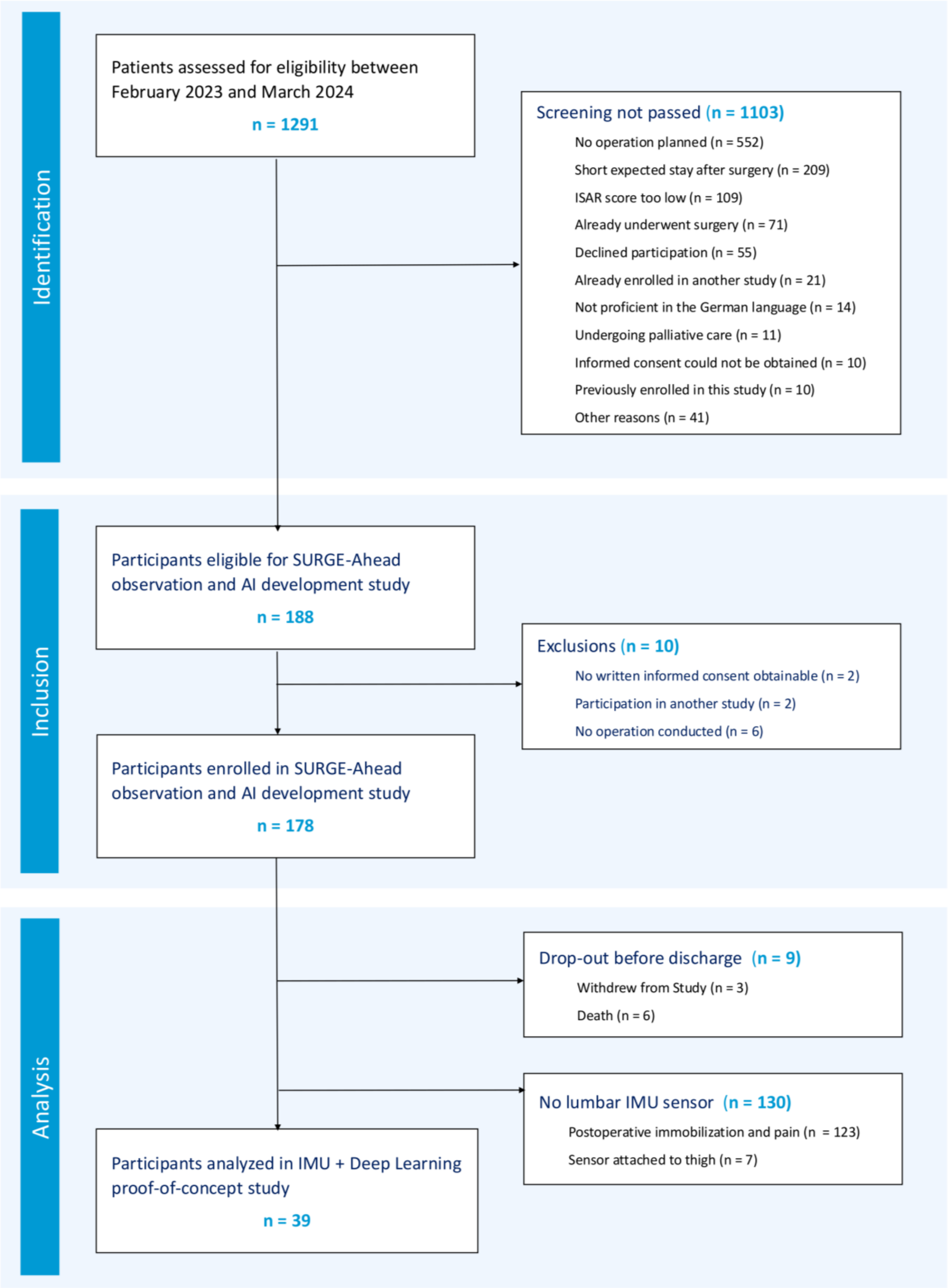

Figure S2: Sensor application and orientation. +acc_x, +acc_y, and +acc_z indicate positive acceleration along the respective x, y, and z axes.

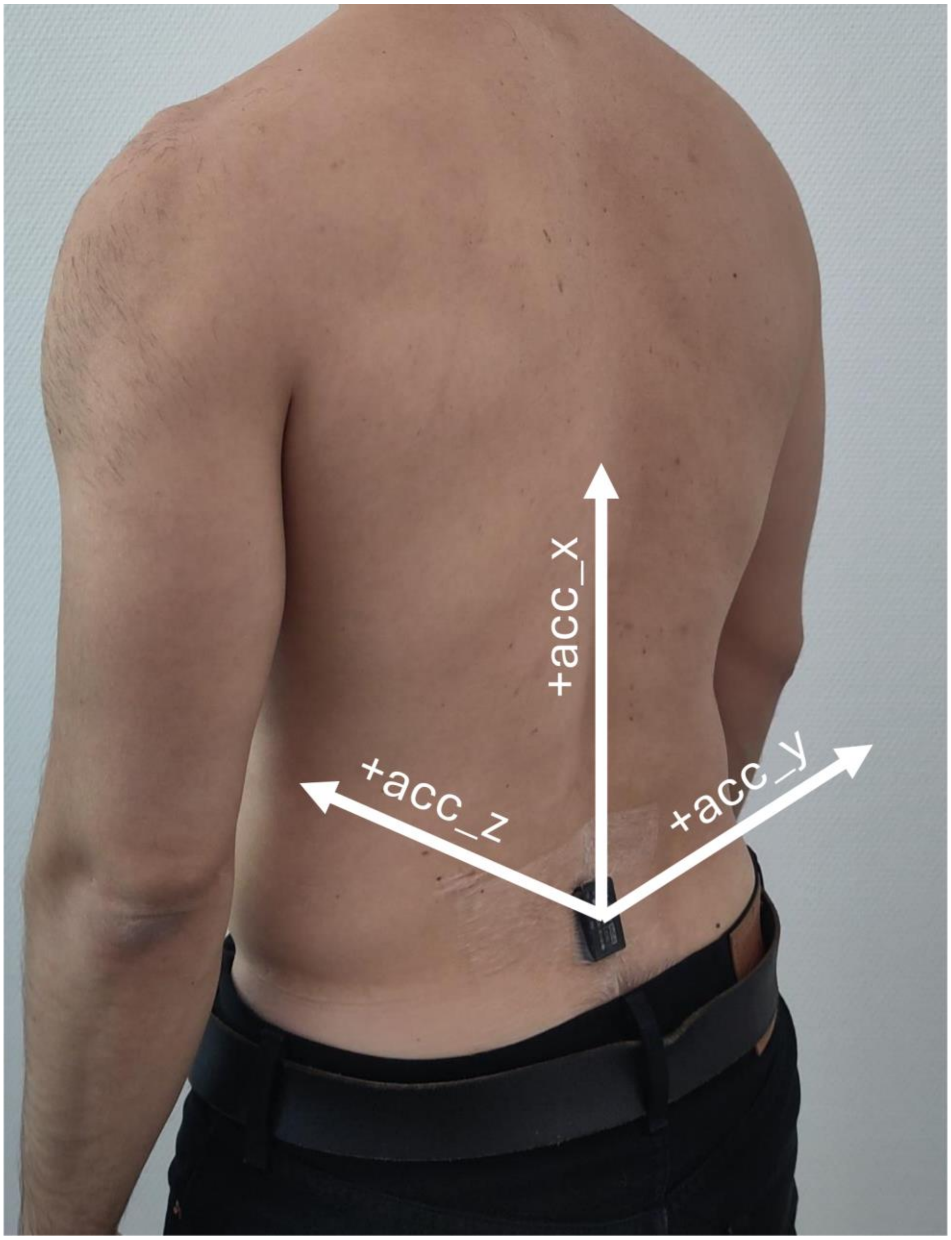

Supplement: Supplementary file 1 [file sensors-25-05021-s001.zip › supplementary figures.pdf]
